# "I prefer dying fast than dying slowly", how institutional abuse worsens the mental health of stranded Syrian, Afghan and Congolese migrants on Lesbos island following the implementation of EU-Turkey deal

**DOI:** 10.1186/s13031-018-0172-y

**Published:** 2018-09-05

**Authors:** Christos Eleftherakos, Wilma van den Boogaard, Declan Barry, Nathalie Severy, Ioanna Kotsioni, Louise Roland-Gosselin

**Affiliations:** 1 Médecins Sans Frontières, Operational Centre Brussels, Mythimnis 27 A, Kypseli, Athens, Greece; 2 Médecins Sans Frontières, Operational Research Unit, 68 rue Gasperich, L-1617 Luxembourg City, Luxembourg; 3 Médecins Sans Frontières, Operational Centre Brussels, Theofanous 19-21, Ampelokipi, Athens, Greece; 4 Médecins Sans Frontières, Operational Centre Brussels, Medical Department, Rue de l’Arbre Bénit, 1050 Brussels, Belgium

**Keywords:** Institutional abuse, EU/Turkey-deal, Migrants, Refugees, Asylum seekers, Greece, Lesbos, Mental health, Traumatic stress

## Abstract

**Background:**

In 2015 and early 2016, close to 1 million migrants transited through Greece, on their way to Western Europe. In early 2016, the closure of the “Balkan-route” and the EU/Turkey-deal led to a drastic reduction in the flow of migrants arriving to the Greek islands. The islands became open detention centers, where people would spend months or years under the constant fear of being returned to Turkey.

Syrians were generally granted refugee status in Greece and those arrived before the 20th of March 2016 had the option of being relocated to other European countries. Afghans had some chances of being granted asylum in Greece, whilst most migrants from the Democratic Republic of Congo were refused asylum.

In a clinic run by *Médecins sans Frontières* on Lesbos Island, psychologists observed a deterioration of the migrant’s mental health (MH) since March 2016. In order to understand the MH needs for this stranded population it was essential to explore how, and by what factors, their mental health (MH) has been affected on Lesbos Island due to the EU/Turkey-deal.

**Methods:**

This was a qualitative study in which eight service providers’ interviews and 12 focus group discussions with male and female Syrian, Afghan and Congolese migrants in two refugee camps on Lesbos Island. Thematic-content analysis was manually applied and triangulation of findings was undertaken to enhance the interpretation of data.

**Results:**

Three main themes were generated: 1) Institutional abuse, 2) Continuous traumatic stress (CTS) and 3) MH service provision. Institutional abuse was expressed by inhumane living conditions, lack of information in order to make future decisions, humiliation and depersonalization. This led to CTS that was expressed through being in a state of permanent emergency under lack of protective measures. Delays in appointments, lack of psychiatric care and differences in MH perceptions amongst migrants highlighted the provision of MH services.

**Conclusion:**

The EU/Turkey-deal reduced migrant flows at a very high price. Decongestion of the camps and the elimination of institutional abuse is urgently needed to reduce CTS and improve migrants’ MH.

## Background

In 2015, Greece had, with over 800,000 arrivals, the highest influx of migrants entering into Europe [1]. The closure of the “Balkan-route” and the EU/Turkey-deal on the 18th of March 2016, led to a drastic reduction in the flow of migrants arriving in Greece. Those that continued arriving, although smaller in numbers [2], were blocked on the islands, under the principle that they should be returned to Turkey. The islands rapidly became open detention centers, where people would spend months or years battling for their right to stay and receive protection in Europe and under the constant fear of being returned to Turkey [3].

Syrians were generally granted refugee status in Greece [4] and those arrived before the 20th of March 2016 had the option of being relocated to other European countries [5]. Afghans had some chances of being granted a refugee status in Greece, whilst most migrants from the Democratic Republic of Congo (DRC) were refused asylum and being processed, for a return to Turkey. Irrespective of one’s nationality, a defined “vulnerability status” was a key factor to define whether a transfer to the mainland in Greece was granted. This was considered to be a positive step into the asylum seeking process. Lastly, the so called high or low asylum recognition rate (Table 1) was another factor influencing the asylum seeking process. Despite the different recognition rate for the Syrian, Afghan and Congolese migrants, it did not affect as such the asylum seeking process as such for any of them and consequently prolonged their stay of all of them on the island.

**Table 1:** Asylum recognition rate and vulnerability status on Lesbos Island, Greece, 2017

Asylum seekers stranded on Lesbos Island have three main options: a) obtaining a refugee status in Greece, b) deportation to Turkey, c) deportation to their country of origin. Different nationalities were categorized under the so-called “high (Syrian and to a lesser extend Afghans) and low (Congolese) asylum recognition rate”. Countries with a low asylum recognition rate were for the most part detained upon arrival and almost exclusively had their asylum claim rejected. Much of their fate depended on their nationality rather than their risk of persecution. Vulnerability status according to the categories as defined in Greek law influences who is obliged to stay on the island for the asylum process. All people arriving on the islands have to undergo a vulnerability screening before their asylum seeking claim will be examined by the Greek authorities. Through vulnerability identification, migrants are allowed to move to the Greek mainland and have their asylum claim examined there, while gaining better access to services they might require. However, the process for the identification of vulnerability was subject to prolonged delays and continuous modifications. Therefore large numbers of migrants were not recognized as vulnerable, and consequently remained stranded on the island.	

It is well documented that people who have fled from war-torn countries and who have experienced traumatic events during their journey are known to have increased mental health (MH) problems [6]. A systematic review from Fazel et al. showed that long term displacement and prolonged stay under inadequate living conditions can have negative consequences on the MH of migrants [7]. Studies on MH problems among migrants around the world and Europe in particular, are more related to whether migrants have just arrived in Europe [8], or have settled in destination countries [9–12]. To our knowledge there is a paucity of information about MH among migrants being stranded in a slow transit country such as Greece.

Since 2015, Médecins Sans Frontières (MSF) has been providing MH services to migrants stranded on Lesbos Island as the availability of these services are extremely limited. Migrants in Greece have by law, free access to public health care. However the provision of MH services in the only hospital on Lesbos, being staffed with only one psychiatrist, is extremely limited. MSF was one of the actors providing MH services to migrants on Lesbos Island. MH service provision has been challenging due to the increased demands and the large number of different nationalities from different cultural backgrounds. An October 2017 MSF report showed that migrants on Lesbos Island are in a state of MH emergency [13]. In order to be able to have better adapted MH services for this diverse and unique migrant population, it was deemed to be essential to explore factors impacting the MH of the Syrian, Afghan and Congolese migrants, as the largest nationality groups, stranded on Lesbos Island due to the EU/Turkey-deal.

## Methods

### Study design

A prospective qualitative study was conducted as the study aim required an exploratory approach [14]. Key informant interviews (KIIs) with health and social care providers were performed, while focus group discussions (FGD) were conducted with Syrian, Afghan and Congolese male and female adult migrants between July and August 2017 (Table 2).

**Table 2:** Syrian, Afghan and Congolese male/female migrant participants in FGDs, Moria and Kara Tepe camp, Lesbos Island, Greece, 2017

Code Name	Interview Type	Nationality	Gender	Average age	Camp name	Language	Number of participants in each FGD
SMM1	FGD	SYRIAN	Male	35	Moria	ARABIC	3
SMM2	FGD	SYRIAN	Male	40	Moria	ARABIC	3
SWKT	FGD	SYRIAN	Female	30	Kara Tepe	ARABIC	6
SMKT	FGD	SYRIAN	Male	45	Kara Tepe	ARABIC	9
AMM	FGD	AFGHAN	Male	45	Moria	FARSI	7
AMKT	FGD	AFGHAN	Male	40	Kara Tepe	FARSI/URDU	7
AWKT1	FGD	AFGHAN	Female	30	Kara Tepe	FARSI/URDU	7
AMKT2	FGD	AFGHAN	Male	40	Kara Tepe	FARSI	6
CMM	FGD	DRC	Male	30	Moria	FRENCH	7
CWM1	FGD	DRC	Female	30	Moria	FRENCH	6
CWM2	FGD	DRC	Female	30	Moria	FRENCH	6

*FGD* Focus Group Discussion, *DRC* Democratic; First letter stands for nationality (Syrian, Afghan, Congolese), Second letter stands for gender (Male, Female), Third letter stands for camp (Moria, Kara Tepe)

### Study setting

Lesbos Island is one of the four islands in Greece very close to the Turkey’s border and therefore made it one of the main entry points into Europe. During November 2015, a few months before the implementation of the EU/Turkey-deal, migrants where arriving on Lesbos Island at the rate of 3300 per day [14]. This however decreased dramatically after the introduction of the EU/Turkey-deal [15] although overcrowding remained a tangible problem.

### Specific study setting

Lesbos Island has the capacity to host 3300 migrants in two camp sites: Moria and Kara Tepe.

Moria is the largest camp on the island with a capacity to host approximately 2300 migrants. It is also one of the four reception and identification centers (RIC) that were created on the Greek islands after the implementation of the EU/Turkey-deal. All migrants were registered at the RIC and interviewed by the Greek Asylum Service (GAS) or EASO (EU’s asylum support office). Prolonged delays in the asylum procedures and the concontinued new arrivals, albeit lower in number, resulted in overcrowding. In addition, lack of funding resulted in many actors ending their service provision, resulting in a worsening situation for the migrants. At the time of the study approximately 3000 migrants were in Moria.

Kara Tepe camp, with a capacity for 1000 people, is managed by the municipality of Lesbos. It is a camp dedicated to accommodate vulnerable migrants such as families, and people with disabilities. After a migrant has been registered at Moria she or he or the family, can be transferred to Kara Tepe, if there is available space.

### Study population and methodology

The study population was Afghan, Syrian and Congolese migrants residing in Kara Tepe and Moria camp on Lesbos Island, and health-and social care providers working with MSF for migrants for at least one year in Greece (Tables 2, 3). Syrian and Afghan migrants represented the largest migrant population who had arrived on Lesbos Island since 2015, and remained high at the time the study was conducted. Since March 2016, there was an increase in migrants from DRC arriving on the island [16], which made the Congolese being the highest represented population among the sub-Saharan Africans.

**Table 3:** Key informant participants: Médecins Sans Frontières’ health providers, Lesbos Island, Greece, 2017

Code Name	Gender	Working with migrants in Greece
KI1	Male	5 years
KI2	Female	2 years
KI3	Female	3 months*
KI4	Male	2 years
KI5	Male	2 years
KI6	Female	2 years
KI7	Male	2 years
KI8	Male	1 year and 5 months
KI9	Male	4 years

*KI* Key Informant

*KI3 was included, despite only 3 months working with migrants, in order to have a better gender balance. Health Care providers were; psychologists, cultural mediators and social workers

Inclusion criteria were Syrian, Afghan and Congolese male and female migrants, of at least 18 years and residing in Kara Tepe or Moria camp on Lesbos Island for at least three months before the study was conducted.

The recruitment of the migrants was conducted by using purposive and convenience sampling. The MSF health promotion (HP) team who operated inside Kara Tepe and Moria camp on Lesbos Island identified participants after having been informed clearly about the inclusion criteria. The aim of the study was explained to potential participants in detail in order to avoid other expectations such as obtaining help in their asylum process. Potential participants were however offered access to MSF psychologists and its MH services. Men and women were separately recruited. For both gender groups it was important to have different age categories and family compositions such as; being alone or with family members in order to stimulate the expression of different perceived concerns. For those who were interested, and who matched the composition of the group, contact details were obtained by the HP team and appointments were given. The principal investigator (PI) recruited purposively directly the key informants (KIs).

### Data collection

KIIs and FGDs were conducted between the 1st of July and the 15th of August 2017. The PI performed the KIIs in Greek, while using an interview guide to allow flexibility, in the MSF clinic. This interview guide was designed by experienced people working in this context together with senior qualitative researchers and got mildly adapted after having it pre-tested among similar profile participants. Written informed consent was obtained first and all KIIs, lasting approximately 30-40 minutes were audio recorded and additional notes were taken by the PI.

Migrant participants were re-contacted by the HP team regarding the meeting point and the time of the FGDs. As the FGDs took place in an MSF office, specifically used for confidential conversations outside the camps, the participants were transported by an MSF car and accompanied by an HP until the MSF office. Before the FGDs, coded oral informed consent was taken on an audio recorder, as written informed consent was too sensitive among this population and therefore not appropriate for obtaining consent. All FGDs were conducted by the PI through the aid of cultural mediators (CMs) who translated from the participant’s local languages (Arabic, Farsi Urdu and French) into Greek or English and vice versa. The aiding CMs were selected for their known translation and cultural mediation skills during their daily work in the MSF clinic. Two of the FGDs were conducted directly in French by an experienced qualitative researcher who is fluent in French and co-investigator. The PI was present during these FGDs and took notes. All FGDs were audio recorded. Each FGD, approximately lasting 60 min, was conducted using a flexible interview guide which allowed for probing. Notes were taken by the PI and the CM. After each interview and discussion, a debriefing took place with the CM in order to make sure that socio-cultural specifics were well captured. The participants in the FGDs were asked to use code names instead of their own names. All participants who seemed to be in distress were offered a pause and if necessary a consultation with a dedicated psychologist: this occurred two times.

### Data analysis

Data analysis was conducted following the data collection by the PI and co-investigator during August 2017. For the KIs, all interviews were transcribed in verbatim from Greek into English by the PI. All KIs received a copy of their transcript for verification. After its return and correction, all transcripts were anonymized before analysis. No specific positions of the KIs are mentioned as this could identify certain participants. For the FGDs, the CMs together with the PI, first re-listened to the audio-recordings to re-check the completeness of the translation from the local language into Greek prior to transcribing into English by the PI. All verbatim accounts were anonymized before analysis.

Transcripts were analyzed manually by the PI and co-investigator using the thematic content analysis by Green and Thorogood [14].

This method means that codes and themes were attached to statements from the transcripts in order to structure the data. Continuous reflection on data including re-discussing themes among two co-investigators and trying to build relationships between them was part of the contextualizing and reforming subthemes until three major themes: institutional abuse, continuous traumatic stress and MH service provision emerged within the existing theory and objectives of the study. Investigator triangulation [14] of the findings among the PI and co-investigator, in addition to data triangulation through the analysis of KIIs and event and site observations, ensured the validity of the data analysis such as separate visits in both camps.

### Ethical considerations

The fact that asking migrants for participation in this study, which could potentially raise false expectations concerning their asylum procedures, was discussed and clearly explained to the participants prior to the FGDs. Secondly, in order to mitigate the unforeseen possibility of participants becoming distressed, anxious or mentally worse during the FGDs, psychologists were on stand-by for providing MH services in case this was needed.

## Results

This section will show the results, in three main themes, which were generated by using the earlier, explained thematic content analysis [14]. The three themes that emerged impacting the MH of migrants on Lesbos Island were the following: 1) Systemic or institutional abuse, 2) Continuous traumatic stress (CTS), 3) MH service provision.

In order to appreciate these three emerged themes from the descriptions of the participants, we provide a brief definition as an introduction to each theme heading prior to providing the participants accounts.

### Systemic or institutional abuse

Researchers and organizations define specific forms of institutional or systemic abuse [17, 18]. A general definition of systemic or institutional abuse could include the destruction of a person’s self-esteem and his sense of safety by installing fear and not providing safety including proper living conditions, depriving people from information in order to make future decisions and by systematically insulting, humiliating and de-personalizing through the difference of power in the relationship [19]. In the literature, systemic or institutional abuse is often described in institutions for elderly [20], or patients in mental health institutions [21].

#### Fear and un-safety: “… the Arab had a big knife (…) everyone that was coming closer they cut them…”

Absence or reluctance of security maintenance inside the camp’s premises results in daily fear and insecurity. Frequent burning of premises and violent clashes amongst migrants or between migrants and the police often led to the interposition of the riot police. Migrants express fear in going out at night, especially women.

*“Even to the toilet (…) we go all together. I am afraid for my daughters, there are many young men, the camp is not as safe as it was in the beginning”. (SWKT).*

*“We got very scared. We lock ourselves in the containers but with the fires we were scared that we will burn in here, we thought we will die in here”.* (CWM1).

#### Living conditions: “Moria is for animals”

Whilst some of the infrastructure in both camps has improved since 2015, living conditions in Moria have actually worsened in 2017, mainly due to the fact that it was designed for short stay but ended up accommodating migrants for a prolonged period of time. In addition, dramatic overpopulation, and the reduction of actors due to lack of funding has reduced the access to basic services such as medical provision. As a result, the basic daily living conditions were described as inhumane. Overpopulated iso-boxes and tents causing lack of privacy was a repeated theme.

*“The room in the iso box is full of people, it’s even hard to breathe so if you stay there you wait for everything*”. (CWM).

*“…and they [RIC] put me in a tent with other 200 people to register me, now for more than 8 months…”(*SMM).

#### Uncertainty and lack of information to make future decisions: “*They are keeping us in darkness”*

Slow and constantly changing asylum procedures, not even understandable by service providers, created a high level of uncertainty. Inadequate and confusing information is present throughout the whole asylum processing.

*“Things are changing every day even for us, we have to be updated week by week if not twice a week, sometimes people know more than us.” (KIHP6).*

“*I see people, they arrive and they are moved, but we are the ones staying here, while we are the ones that arrived many months ago” (SMKT).*

*“There is no organization to provide any information” (AMM2).*

#### Intimidation, insults, de-personalization: “…You are only addressed with two words: “no” and “wait”…”

Elimination of each individual’s identity was quoted by many migrants while the response to questions regarding information about their cases or complaints regarding living conditions was usually answered with another insult.

*“…so they see us like refugees, like animals, like we don’t know how to live…” (AWKT1).*

*“I expected people to welcome me, they will know what I’d gone through, but instead they [camp service providers] call me “hey…you” and put me in a tent…” (SMM1).*

*“…when I see people on the barbed wire waiting for food what is the difference with me and sheep? When I see the police beating people in order to wait in line….” (AMM1).*

### Continuous traumatic stress (CTS)

The term CTS was first developed around 1980 by MH professionals working in South Africa [22]. It provides a way to describe the consequences of current conditions in which there is an ongoing danger in the present or future instead of having past traumatic experiences. Main characteristics of CTS can be a) a state of permanent emergency, b) preoccupation with threats on present and future, c) absence of protective measures [23].

#### Permanent emergency “there is no day without a fight”

Continuous tensions and fights inside the camps create a permanent state of emergency. A Syrian man who spent already 13 months in Moria experienced several severe insecurity incidents.

*“I have seen 5 fires and 5 funerals” (SMM2)*.

*“Every night fires broke out in Moria and we were running in the woods with our packs to save ourselves. We didn’t have a single day of calmness. My clothes, they are still in the bags since 1 year” (AWKT1).*

#### Preoccupation with realistic threats on present and future: “*They gave us two choices, you stay here or you go back”*

Most migrants focus on the realistic threats of the present and the future instead of focusing on their traumatic past, including the fear for deportation. A Syrian man from Moria described his desperation regarding the possibility to be deported in Turkey. In addition, those having received their refugee status have new emerging sets of threats.

*“I cannot stand it, there are times that I get disappointed that I will never see my children [in Germany] I will never manage to hug my daughter” (AWKT1).*

“*They announced that whoever gets a refugee status will have six months until the money will stop. I have all the necessary documents. What am I going to do? Even looking for job is very hard, I see the locals most of them are unemployed” (AWKT1).*

*“I wonder how Turkey is a safe country for me (…) the Turkish bombarded my own village, how is Turkey safe for me”* (SMTK).

#### Recurrent absence of protective measures

The recurrent absence of protective measures was expressed by participants among them a woman from DRC and a Syrian man referring to the sudden death of a 19 year old (while showing pictures on his phone).

*“When I went to police and told them that this guy threatened us with a knife they told me that when the drugs go off he will be ok again”.*

*“Do you think this guy with this health will die suddenly, he was 19 years old, no smoking, no drugs, his age was only 19 years” (SMM1).*

#### Obsessive pursuit to obtain a vulnerability status: “I went to the doctor in a hospital to change this word [vulnerability] to make it a yes”

A vulnerability status can potentially help in accelerating the asylum procedures, the transfer to a better accommodation or to the mainland through the lift of geographical restrictions. The identification of vulnerability however is subject to constant modification and prolonged delays, and therefore migrants become obsessed with the pursuit of (potentially) necessary documents (Table 4).

*“We wake up we sleep and we wake up with just the EASO interview, our papers, this is our thinking” (SMKT).*

*“So, this despair, many try to manipulate you to get documents, anything that can help their case (…), they want to hold on to something”* (KI2).

*“They all seek for a reason from a doctor to be sick, to have a paper, the thought came also to me (…), I reached there and started to think what reason shall I give(…)”* (SWKT).

**Table 4:** Construct of vulnerability on Lesbos Island, Greece, 2017

A directive that was applied in April 2017 instructed that vulnerable migrants cannot leave the island before their 1st asylum interview. This is practically translated into a minimum of six months of island restriction. During October 2017 the population on Lesbos Island had reached 5800 migrants, a number far beyond island’s reception capacity. Due to this, it was temporarily allowed to the transfer of 1100 vulnerable migrants onto mainland lifting their geographical restrictions before the 1st asylum interview. However, immediately after the transfer, the directive of April 2017 was reinstated. The un-clarity and rumors around the advantages of being vulnerable makes migrants obsessively preoccupied with the pursuit of a vulnerability document. This results migrants living in a state of permanent emergency. Besides this, project staff identify that vulnerability status does not always guarantee a better treatment. For the migrants, it seems that this vulnerability obsession is a copying mechanism which keeps their hope alive, while for the authorities is nothing more than a way to facilitate and manage the overpopulation on the island.	

#### Isolation, cognitive impairment, substance abuse, self-harm and suicide attempts: “*if I don’t drink alcohol I can’t sleep”*

Research in different settings indicates that in some instances traumatic stress can be a risk factor for more severe psychopathological symptoms [24, 25]. Current conditions in the camps of Lesbos can be a risk for similar symptoms. KIs expressed their concern regarding the increasing suicidal rate, which was also expressed by many migrants. In addition, deterioration of cognitive function and substance abuse was a repeated theme.

#### Self harm

*“They harm themselves by cutting to show their pain that they are desperate and can’t take it anymore in fact it gives relief to the “pain” in the head”* (CWM1).

#### Substance abuse

“*The difference that I have noticed is that all people when they first arrived were healthy, now they have started smoking or drinking, they came for a better life and they ended up alcoholics”(AMM1).*

#### Suicide attempts

“*My husband committed suicide 3 times. All these months I am here I have become so angry that I harm myself” (AWKT2).*

#### Cognitive impairments

*“When you are attending Greek lessons you feel that your mental ability or capacity is low, I have two university degrees, it is completely different now…”* (SMM1).

#### Isolation

“….*he does not want to be social anymore, he just sits and thinks*…” (AMKT).

### MH service provision: “… so, I am a psychologist, how do I treat this?”

Effective provision of MH in terms of quantity and quality was recurrently discussed and being expressed by: a) Delays in appointments, b) Lack of psychiatric care, c) Different perceptions about mental health.

#### Delays in appointments: “You go again and they don’t see you”

“*They give me an appointment (psychiatrist at Public Hospital) 4 months later. I will become crazy after that long” (AWKT2).*

#### Lack of psychiatric care:

“*There’s one psychiatrist at the hospital (public) and one working in private, on Lesbos…” (KI1).*

*“…things are getting worse, suicidal attempts, self-harm. The past three weeks we have had like 15 people on that list [psychiatric care], (….) when usually it’s about 5” (KI3).*

#### Perception of mental health: “Is there a solution for a psychologically sick person?”

Different cultures have different perceptions of what mental health care is about. It is a challenge for the current mental health provision to take into account the following quotes,

*“In Afghanistan you have a problem the doctor gives you medication and you go, they don’t have this idea of psychotherapy, they take the pills” (KI1).*

*“…what they [Afghans] are facing is beyond a psychologist. If you use the standards of Europe all Afghans will need a psychologist”. (AMKT).*

## Discussion

The implementation of the EU/Turkey-deal in the 18th of March 2016 [3] resulted in a dramatic reduction of migrant flows but with a very high cost on migrant’s MH. MH care needs have therefore increased and became more complex. Yet, MH service provisions have not adapted to this, and remain inadequate due to lack of resources and the difficulty to offer this more complex MH care appropriate for the diversity of migrant populations. In addition, independently of MH service provision, current traumatic stressors were reported by most migrants, which led to a prolonged suffering. To our knowledge this was the first qualitative study that was conducted among migrants being stranded in a contained space of a country perceived by most as a transit destination in Europe, and what factors influenced their mental health.

In our findings three main themes were generated: institutional abuse, CTS and lack or inefficiency of MH service provision, which merit attention.

### Institutional abuse

Research on institutional abuse and the variety of settings in which it is taking place is still ongoing with different approaches towards its definition [26]. In our research, the term institution refers to the setting where psychological abuse can be expressed and analyzed in the context where, “the abuser holds a position of trust and authority over the abused one” [27]. This definition would include the camps on Lesbos Island. From our findings, the main camp authorities, who are responsible for the provision of accurate information on asylum procedures, security and proper living conditions did not fulfill their protective role. On the contrary, as was repeatedly expressed by most of the participants their behavior was more of an abusive kind.

Chetail and Brauenlich suggest that stranded migrants, independent of nationality are commonly subjected to a wide range of psychological abuses and violations committed by a range of actors and institutions [28]. Literature on institutional abuse frequently includes groups of people that are in need of care like those within a psychiatric institution [29] or within institutional care for the elderly [30]. It is commonly characterized “by the systemic destruction of a person’s self-esteem through psychologically abusive tactics” [31]. Although psychological abuse inside psychiatric institutions or institutions for the elderly cannot fully account for the devastating life conditions of the refugee camp, the vast majority of migrants expressed the dependency on governmental and camp authorities.

Migrants reported being left with no reference point to provide answers regarding their prolonged stay and the daily struggle in inhumane living conditions. Camp authorities are unable to provide sufficient answers or solutions regarding the absence of proper protection, their future options, the humiliation of waiting in lines for basic services like water and food provision and the constant fear of deportation. We therefore consider that under the EU/Turkey-deal abusive relationships are formed, where the perpetrators are the camp authorities like EASO, GAS and police. On the other hand, the victims, who are the migrants, are obliged to spend a prolonged period of time in a camp environment that systemically destroys their self-esteem. 

### Continuous traumatic stress (CTS)

It is argued that the effects of victimization through psychological abuse and the presence of continuous stress can lead to severe trauma [32]. Displaced populations by persecution or warfare find themselves living in contexts where the traumatic stressors are continuous [33], and/or in a state of permanent emergency. A study that was conducted in Serbia among migrants travelling along the Balkan route, showed that out of 992 migrants who attended MH consultations, 383 had experienced at least one but even up to three traumatic events during their journey since leaving their country of origin [34]. Continuous fears of being deported to Turkey or the country of origin, continuous pursuit of a vulnerability status, continuous threats on the personal sense of safety are some of the examples migrants experience on Lesbos Island. These ongoing and realistic threats demonstrate a consistent focus on present and future stress.

In the literature there are different opinions whether CTS can actually lead to more serious psychopathological symptoms, like post traumatic stress disorder (PTSD), major depression or anxiety disorders [35]. A study from Israel connects CTS with the development of PTSD, complex PTSD or acute stress disorder [36]. Another study by Bleich et al. in a general population in Israel found a moderate psychological reaction under repeated traumatic experiences. They suggest that prolonged exposure to traumatic events may increase the level of severity of other types of psychopathology more than it increases the risk for PTSD [37].

In our study setting, the participants demonstrated a variety of symptoms under prolonged traumatic stress: self harm, substance abuse, suicide attempts, cognitive impairments, and isolation. It is unclear whether these symptoms are directly connected with CTS. Recent research has mainly focused on examining psychopathological reactions under continuous traumatic stress comparing factors such as type and duration of exposure, geographical context and ethnical identity [38, 39]. Further research is needed in order to demonstrate whether individual perceptions, specificities of the camp living condition, characteristics of traumatic stress and cultural perspectives are connected with the above symptoms.

### MH service provision

Individualized MH provision is difficult due to the complexity and the lack of experience in similar settings were current traumatic stressors are always present. Since the setting of our research is relatively new, the adaptation of MH services for migrants needs is challenging and often blurred by the various cultural perceptions each nationality has.

People experiencing traumatic events often search for explanations and meanings of the context where such events are taking place. Cultural beliefs concerning misfortune or loss are shaping individual’s attributions. This often leads to a return to their cultural and traditional explanatory systems that dissociate from western conceptions [40]. The complexity of providing such context-specific MH care can lead to prolonged untreated psychological problems which enhance the risk for the development of psychiatric symptoms. Lack of resources in the delivery of proper psychiatric care is often directing migrants to devastating alternatives such as self-harm, substance abuse or self- medication. The need for MH interventions for migrants and refugees has been stressed by studies in different contexts whilst also models for adaptation of MH services to migrant’s characteristics have been proposed [41].

The need for MH service provision for migrants, and their efforts to provide access to MH services in extremely harsh conditions, was described by the MSF medical team in Croatia [42]. This was also characteristics by the high numbers of consultations in MSF Lesbos clinic and the reports of professionals. Despite the differences in context, the need for creating a safe space away from the daily living stressors is a common goal for service providers. Continuous stress is a characteristic of inadequate living conditions. However, when it is combined with social and physical impairments, the request for MH interventions is an important determinant for the MH of migrants. In order to provide standards for MH service provision, thresholds should be set for identifying those in need, taking into account the cultural norms through the participation of communities in the development of MH action plans [43].

### Policy implications

The decision of the EU Commission and the Turkish and Greek governments to implement the EU/Turkey-deal in an effort to discourage migrant flows from Turkey to Europe has come with a very high price. Other such similar deals with Libya, Afghanistan and West Africa are likely to have similarly devastating effects on the life and health of migrants.

It has been well documented that, in some cases, “the symptoms of CTS, tend to diminish dramatically or completely resolve when they are no longer within harm’s way” [44].

Possible solutions to end CTS of the migrant population on Lesbos Island require an immediate stop of the institutional abuse, decongesting the islands by transferring people to the mainland, by improving the living conditions, and providing access to information and clear asylum procedures for everybody.

### Strengths and limitations

Strengths of this study were that the verbatim transcripts of the KIs were re-evaluated by themselves prior to analysis. The study included participants from both camps as well as KIs which allowed triangulation of the data. The migrants were recruited by the HP-team who know the population well, and could guarantee that the inclusion criteria were maintained and an appropriate mix in terms of age and status were grouped. In addition, the CMs that were present during the FGDs and GIs are not only translators but familiar with the respective cultures of the interviewees. Hence, misinterpretations of the participants’ accounts were minimal. On the other hand, using CMs created a second layer of communication, and although post FGD debriefings took place, it is possible that some information was missed. However, quality assured as much as possible through the reevaluation of the translation by the same CM who translated during the FGD.

Other possible limitations include: results refer to the particular period in which the study was conducted due to the constantly changing context of migrant population. It was also not always possible to recruit a group of 6-12 participants .The PI, being Greek, was in some occasions associated with the Greek government, which provoked in some participants a hostile response, which potentially may have biased their perceptions.

The fact that the PI and co-investigator were identified as MSF staff may have created expectations amongst the participants, and consequently created a response bias. However, this was surpassed through the detailed explanation of their role and the objectives of the study, reinforcing the knowledge that no direct benefit could be obtained by their participation.

A serious security incidence in Moria, which occurred at the same time as the FGDs might have influenced participants’ responses. Lastly, one KI out of eight had less than one year of working experience with migrants in Greece.

## Conclusion

This first qualitative study showed that institutional abuse creates an environment where traumatic stressors are continuous, while the provision of adapted MH services for this specific migrant population is inadequate or insufficient. In order to avoid future repetition, institutional abuse needs to be addressed while living conditions needs to be improved allowing for a cessation of CTS. Lastly, mental health service provision need a more adapted and flexible approach, by taking into consideration the context of Lesbos Island. The development of an intervention, including the appropriate human resources, which targets parameters such as: asylum procedures, security and living conditions, which maintain the continuity of stress, are essential and urgently needed. In addition, understanding cultural diversity and perception of migrants for MH will facilitate the better adjustment of the MH care provision.

## Abbreviations

CM: Cultural Mediators
CTS: Continuous Traumatic Stress
DRC: Democratic Republic of Congo
EASO: European Union Asylum Support Office
ERB: Ethical Review Board
EU: European Union
FGD: Focus Group Discussion
GAS: Greek Asylum Service
GI: Group Interviews
HP: Health Promoters
KI: Key Informant
KII: Key Informant Interview
MH: Mental Health
MSF: Médecins Sans Frontières
PI: Principal Investigator
PTSD: Post Traumatic Stress Disorder
RIC: Reception and Identification Centers
UNHCR: United Nations High Commissioner for Refugees
